# PMQR genes *oqxAB* and *aac(6′)Ib-cr* accelerate the development of fluoroquinolone resistance in *Salmonella typhimurium*

**DOI:** 10.3389/fmicb.2014.00521

**Published:** 2014-10-02

**Authors:** Marcus H. Wong, Edward W. Chan, Li Z. Liu, Sheng Chen

**Affiliations:** ^1^Food Safety and Technology Research Centre, Hong Kong Polytechnic University – Shenzhen Research InstituteShenzhen, China; ^2^State Key Laboratory of Chirosciences, Department of Applied Biology and Chemical Technology, The Hong Kong Polytechnic UniversityKowloon, China; ^3^Department of Microbiology, The Prince of Wales Hospital – The Chinese University of Hong KongShatin, China

**Keywords:** *S. typhimurium*, ciprofloxacin resistance, ACSSuT R type, *oqxAB*, *aac(6*′)*Ib-cr*

## Abstract

Emergence of multidrug-resistant *Salmonella typhimurium* strains, especially the ACSSuT and nalidixic acid R types, has significantly compromised the effectiveness of current strategies to control *Salmonella* infections, resulting in increased morbidity and mortality. Clinical *S. typhimurium* isolates recovered in Hong Kong during the period of 2005–2011 were increasingly resistant to ciprofloxacin (CIP) and antibiotics of the ACSSuT group. Our data revealed that *oqxAB* and *aac(6′)Ib-cr* were encoded on plasmids of various sizes and the presence of these two elements together with a single *gyrA* mutation in *S. typhimurium* were sufficient to mediate resistance to CIP. Acquisition of the *oqxAB* and *aac(6′)Ib-cr* encoding plasmids by *S. typhimurium* caused a fourfold increase in CIP minimal inhibitory concentration. Furthermore, the presence of *oqxAB* and *aac(6′)Ib-cr* in *Salmonella* dramatically increased the mutation prevention concentration of CIP which may due to mutational changes in the drug target genes. In conclusion, possession of *oqxAB* and *aac(6′)Ib-cr* encoding plasmid facilitate the selection of CIP resistant *S. typhimurium*, thereby causing a remarkable increase of CIP resistance among clinical *Salmonella* strains in Hong Kong.

## INTRODUCTION

Non-typhoidal *Salmonella* are among the principal bacterial pathogens implicated in food-borne gastroenteritis worldwide (Gomez et al., 1997). Antimicrobial agents are not usually required for treatment in salmonellosis but can be lifesaving in cases of severe or systemic infections, as well as treatment for elderly and immunocompromised patients (Hohmann, 2001). Multidrug resistance in *Salmonella* has been documented since 1980, a representative class of resistant organisms being the ACSSuT resistance type of *Salmonella typhimurium* DT104, which originated in the UK and spread rapidly to the US and other parts of the world (Centers for Disease Control and Prevention, 1997; Glynn et al., 1998; Markogiannakis et al., 2000). Hence fluoroquinolones and the extended-spectrum cephalosporins have become the drugs of choice for treatment of acute gastroenteritis caused by *Salmonella* and other enteric pathogens. Resistance toward quinolone and fluoroquinolone antimicrobials is mainly due to target mutations in quinolone resistance determining region (QRDR) of DNA gyrases (*gyrA* and *gyrB*) and Type IV topoisomerases (*parC* and *parE*), which subsequently prevent drugs from binding (Hawkey, 2003). Although high level fluoroquinolone-resistant *Salmonella* are known to be associated with specific serotypes of *Salmonella* and have been reported in scattered regions around the world, their prevalence remains low. This is probably due to the fact that the process of selection of double *gyrA* and single *parC* mutations is not very efficient. Nevertheless, several lines of evidence have suggested that emergence of multidrug resistant non-typhoidal *Salmonella* strains has significant impact on the effectiveness of current strategies, including reduced efficacy of early empirical treatment to control and manage diseases associated with food-borne infections. These include reduced efficacy of early empirical treatment as well as limited choice of treatment.

Plasmid mediated quinolone resistance (PMQR) genes such as *qnrA, qnrB, qnrC, qnrD, qnrS, qepA,* and *aac(6′)Ib-cr* have been increasingly reported in bacterial pathogens. The *qnr* type PMQR genes bind to DNA gyrase and topoisomerase to block the action of fluoroquinolones resulting in reduced susceptibility to fluoroquinolones (Tran et al., 2005). QepA encodes a MFS-type eﬄux pump which is able to excrete quinolone into extracelluar space (Yamane et al., 2007). AAC(6′)Ib-cr acetylates ciprofloxacin (CIP) and norfloxacin (Robicsek et al., 2006). It is postulated that these PMQR genes are able to contribute to the development of quinolone resistance in these organisms (Cattoir and Nordmann, 2009). Recently, a novel transmissible resistance-nodulation-division (RND) eﬄux pump OqxAB, which mediated resistance to olaquindox, chloramphenicol, nalidixic acid, and elevated minimal inhibitory concentrations (MICs) of other antimicrobial reagents including ampicillin, gentamicin, and CIP (MIC between 0.06 and 0.25 μg/ml), has been identified (Hansen et al., 2007). OqxAB was encoded on an IncXI plasmid, pOLA52, harbored by a swine *Escherichia coli* isolate (Hansen et al., 2004). More recently, OqxAB was reported to be prevalent in organisms isolated from pork and pig farms in China (Zhao et al., 2010; Liu et al., 2011; Wong and Chen, 2012), as well as from human food (18). On the other hand, the *oqxAB* gene has not been found in clinical isolates until recently, when it became detectable in clinical strains of *E. coli*, *Salmonella,* and *Klebsiella pneumoniae* (Kim et al., 2009; Park et al., 2012; Ruiz et al., 2012; Yuan et al., 2012). The functional and clinical significance of this and other PMQR genes such as *qnrA, B, D,* and *S*, which have also become prevalent in clinical *Salmonella* isolates (Cavaco et al., 2009; Ferrari et al., 2011; Kim et al., 2013), remains unclear.

We have previously characterized 239 human clinical *S. typhimurium* isolates recovered from hospital patients in Hong Kong during the period of 2005–2011 for their susceptibilities to fluoroquinolones and other antibiotics and the prevalence of PMQR genes. Two PMQR genes, *oqxAB* and *aac(6′)Ib-cr*, were found to exhibit close relationship with fluoroquinolone resistance in *S. typhimurium*. Approximately 44% of the *oqxAB*-positive *S. typhimurium* were resistant to CIP and around 89% of *oqxAB*, *aac(6*′*)Ib-cr-*positive isolates were resistant to CIP, while only 11% of the *oqxAB*-negative isolates were resistant to CIP (Wong et al., 2013). In the current study, we want to investigate the direct association of *oqxAB/aac(6*′*)Ib-cr* with the development of fluoroquinolone resistance in *S. typhimurium*. We confirm that *oqxAB* and *aac(6*′*)Ib-cr* can mediate rapid development of fluoroquinolone resistance in *S. typhimurium* and could be a contributive factors accounting for the increasing prevalence of fluoroquinolone-resistant *Salmonella* in Hong Kong in recent years.

## MATERIALS AND METHODS

### BACTERIAL STRAINS

239 *S. typhimurium* previously described clinical isolates were included in this study (Wong et al., 2013).

### PCR AND TARGET GENE MUTATION SCREENING IN *S. typhimurium*

The QRDRs of *gyrA* and *parC* were amplified by PCR as previously described (Chen et al., 2007), followed by determination of their nucleotide sequences and comparison to the wild-type *S. typhimurium* LT2 strain to identify target gene mutations in the test strains. The *gyrA* and *parC* sequences of four *Salmonella* isolates, S08-52, S10-9, S05-23, and S05-30, were submitted to GenBank with the accession numbers for *gyrA*, KM504240, KM504241, KM504242, and KM504243 and *parC*, KM513651, KM513652, KM513653, and KM513654. The association of Insertion sequence IS*26* with *oqxAB* was determined by PCR using primers IS*26-*F(5′GCTGTTACGACGGGAGGAG) and oqx-R (5′ GGAGACGAGGTTGGTATGGA).

### CONJUGATION EXPERIMENTS

A conjugative experiment was carried out as previously described (Jacoby et al., 2003) using sodium azide-resistant *E. coli* J53 strain as recipient. Briefly, overnight culture of donor and recipient strains were mixed and collected on a filter, which was subjected to overnight incubation on a blood agar plate. The mixture was then spread on double selective blood agar plates containing olaquindox (128 μg/ml) and sodium azide (100 μg/ml).

### S1-PFGE AND HYBRIDIZATION

S1-PFGE was conducted to determine the size of large plasmids. Briefly, agarose-embedded DNA was digested with S1 nuclease (New England BioLab) at 37°C for 1 h. The restriction fragments were separated by electrophoresis in 0.5 Tris-borate-EDTA buffer at 14°C for 18 h using a Chef Mapper electrophoresis system (Bio-Rad, Hercules, CA, USA) with pulse times of 2.16 to 63.8 s. Phage Lambda PFGE ladder (New England BioLab) was used as DNA size marker. The gels were stained with GelRed, and DNA bands were visualized with UV transillumination (Bio-Rad). Chromosomal and plasmid DNA of *S. typhimurium* strains were transferred and cross-linked onto nylon membrane and hybridized with a DIG-labeled *oqxAB* probe using DIG High Prime DNA Labeling and Detection Starter Kit I (Roche) following manufacturer’s instructions to determine the localization of *oqxAB* and *aac(6*′*)-Ib-cr* genes in *S. typhimurium* genetic materials.

### *oqxAB* CLONING, PLASMID TRANSFORMATION, AND PLASMID CURING

Cloning of *oqxAB* into pTrcHisB (Life Technologies) vector was done by PCR using primers pTrc-oqxAB-F (5′TTACTACTCGAGAATGAGCCTGCAAAAAAC) and pTrcoqxAB-R (5′AGGATCGAATTCCTAGGCGGGCAGATCCTC). pTrc-*oqxAB* was transformed into *S. typhimurium* LT2. Plasmids from clinical strains were extracted by Qiagen Mini-prep kit, electroporated into *S. typhimurium* LT2 and a nalidixic acid and CIP susceptible *S. typhimurium* clinical strain 11–28, and selected on plates containing 32 μg/ml olaquindox. Plasmid curing was performed on clinical *S. typhimurium* strain 10–63 as previously described with slight modification (Sato et al., 2013). The strain was grown in 3 ml LB at 43°C for 2 weeks and selected on plates containing 0, 8, 16, 32 μg/ml olaquindox.

### MUTATION PREVENTION CONCENTRATION (MPC)

Mutation prevention concentration (MPC) of *oqxAB*, *aac(6*′*)Ib-cr* positive, and negative strains was determined as described previously (Gebru et al., 2011, 2012). Briefly, MPC was determined by spreading 1 × 10^9^ cells on LB agar plates containing a range of concentration of CIP: 0, 0.05, 0.1, 0.25, 0.5, 1, 2, 4, 8, 16, 32 μg/ml. Plates containing CIP were incubated for up to 72 h, whereas CIP-free plates were incubated for 24 h. Viable counts on each plate were recorded. MPC was defined as the lowest antibiotic concentration at which no colonies were observed. For each strain, MPC was determined on the basis of the results of at least three independent experiments.

## RESULTS

### PMQRS AND SINGLE GyrA MUTATION MEDIATE DEVELOPMENT OF FLUOROQUINOLONE RESISTANCE IN *S. typhimurium*

To determine if *oqxAB* alone or the combination of *oqxAB* and *aac(6*′*)Ib-cr* can contribute to fluoroquinolone resistance, the effect of interplay between *oqxAB*, *aac(6*′*)Ib-cr,* and target mutations in mediating fluoroquinolone resistance phenotypes in *Salmonella* was studied. Among all *oqxAB* negative *S. typhimurium* organisms, the vast majority of those which exhibited CIP MIC ≤0.05 μg/ml had no mutation in the *gyrA* and *parC* genes. Single amino acid substitution (D87Y or D87N) in GyrA was often detected in strains with CIP MIC between 0.1 and 1 μg/ml. Interestingly, two CIP-resistant isolates (CIP MIC = 1 μg/ml) showed only single mutation at D87Y. Double substitution in GyrA (S83F and D87Y or N) and a single substitution in ParC (S80I) were consistently detected in those with CIP MIC ≥2 μg/ml (**Table 1**). Among all *oqxAB* positive *S. typhimurium* strains, no mutation was detected in *gyrA* and *parC* in strains with CIP MIC ≤0.05 μg/ml; single mutation in GyrA (D87Y or D87N), but not in ParC, was detected in strains whose CIP MIC was between 0.25 and 2 μg/ml. Among all isolates which were positive to both *oqxAB* and *aac(6*′*)Ib-cr*, single mutation in GyrA (D87Y or D87N), but not in ParC, was detected in strains whose CIP MIC was between 0.25 and 2 μg/ml, whereas most of the strains from this category exhibited CIP MIC ≥1 μg/ml. Comparative analysis of mutational and drug susceptibility data of *oqxAB* negative, *oqxAB* positive, and *oqxAB*, *aac(6*′*)Ib-cr* positive strains showed that similar mutational profiles could result in drastically different CIP MIC, depending on whether the organism harbored the *oqxAB* or *oqxAB, aac(6*′*)Ib-cr* genes. Strikingly, simultaneous presence of a single *gyrA* mutation and *oqxAB*, or both *oqxAB* and *aac(6*′*)Ib-cr* genes, was sufficient to produce CIP resistance (CIP MIC = 1 μg/ml); however, double mutations in *gyrA* plus a single mutation in *parC* were required to mediate CIP MIC ≥2 μg/ml when *oqxAB* was absent. Importantly, around 98% of *oqxAB* positive *S. typhimurium* strains harbored mutations in the *gyrA* or *parC* genes, whereas less than 60% of *oqxAB* negative *S. typhimurium* strains had mutations in either or both of these two genes (Data not shown). Taken together, these findings suggest that acquisition of *oqxAB* or *oqxAB*, *aac(6*′*)Ib-cr* by *S. typhimurium* could mediate selection of fluoroquinolone resistance in *S. typhimurium*.

**Table 1:** Presence of target mutations in different level of ciprofloxacin MIC of *oqxAB* positive and negative *Salmonella typhimurium* isolates.

CIP MIC	Clinical *S. typhimurium*								
	*oqxAB*-			*oqxAB*+			*oqxAB+*, *aac(6*′*)Ib-cr*+		
	No. of isolates	*gyrA*	*parC*	No. of isolates	*gyrA*	*parC*	No. of isolates	*gyrA*	*parC*
≤0.05	104	WT/D87N	WT/S80R	1	WT	WT	0		
0.1	24	D87N	WT	0			2	WT	WT
0.25	14	D87N	WT	1	D87Y	WT	1	D87Y	WT
0.5	18	D87N	WT	3	D87Y	WT	3	D87Y	WT
1	2	D87Y	WT	2	D87Y	WT	6	D87Y	WT
2	2	S83F, D87G	S80R	3	D87Y/D87N	WT	35	D87Y/D87N	WT
4	0			0			10	D87Y	WT
8	0			0			0		
≥16	8	S83F, D87G	S80R	0			0		
Total	172			10			57		

### TRANSFERABILITY AND GENETIC LOCATION OF *oqxAB*

**FIGURE 1:**
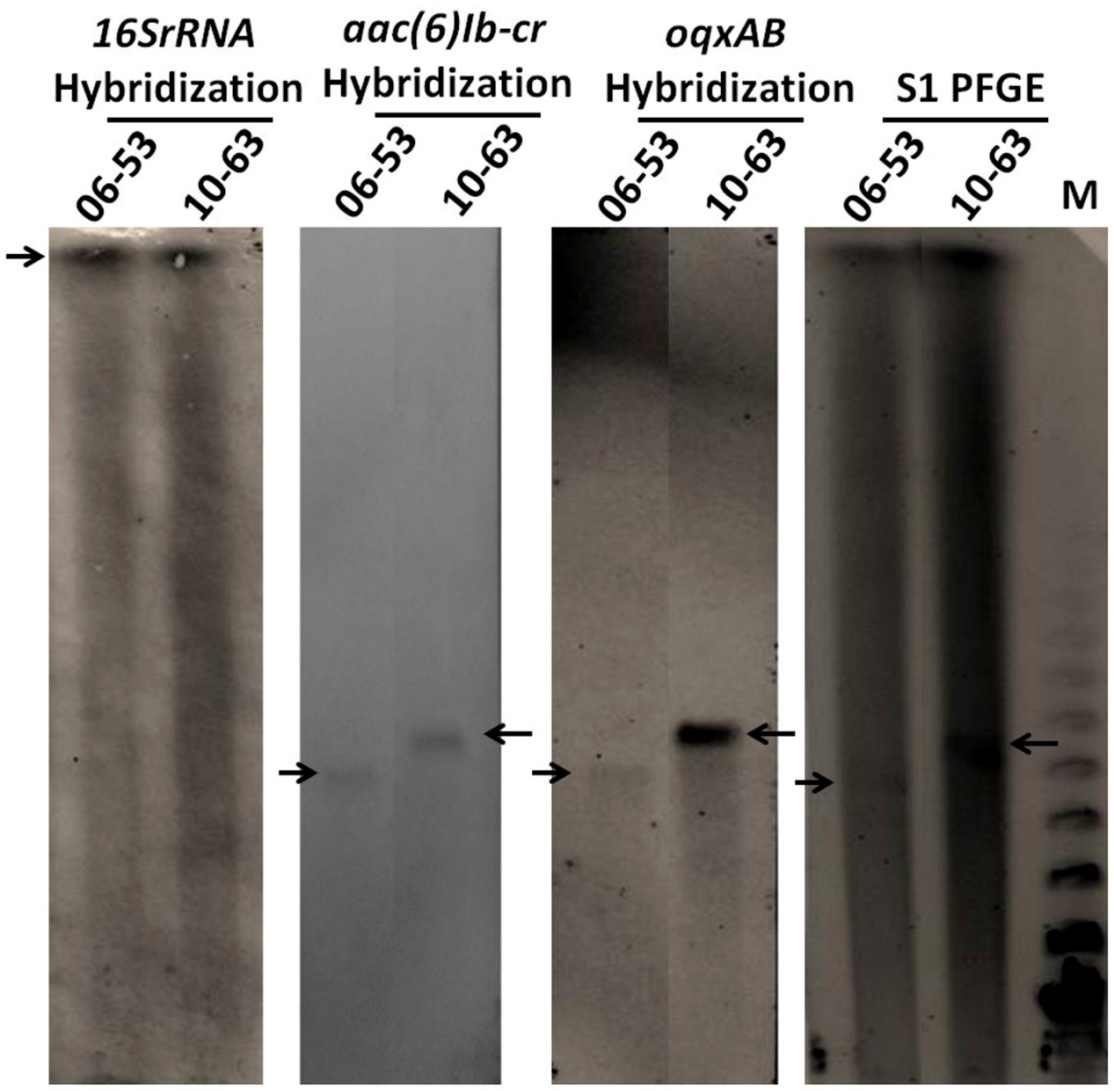
**S1-PFGE and southern hybridization of 16SrRNA, *oqxA*, and *aac(6*′*)Ib-cr* on two oqxAB-positive isolates.** Arrows indicated chromosomal DNA or plasmids harboring *oqxAB* and *aac(6*′*)Ib-cr*. 06–53 and 10–63 are *oqxAB*-positive *S. typhimurium* clinical isolates; M, Lambda PFGE marker.

Thirty randomly selected *oqxAB* positive *S. typhimurium* isolates were subjected to conjugation experiment to determine the transferability of the *oqxAB* gene that they harbored. Surprisingly, none of the *S. typhimurium* strains tested was able to transfer this resistance element to *E. coli* J53 recipient strain through conjugation. S1-PFGE and Southern hybridization were performed on four *S. typhimurium* isolates and it showed that *oqxAB* and *aac(6*′*)Ib-cr* were concurrently present on plasmids of various sizes in these *S. typhimurium* isolates, hybridization results of two of which were shown in **Figure 1**. In all the tested *S. typhimurium* isolates, the *oqxAB* gene was found to be flanked by the IS*26* fragment in a manner similar to that of the pOLA52 plasmid as previously reported (Norman et al., 2008), suggesting that the *oqxAB* gene that was becoming prevalent in *S. typhimurium* could have been derived from the original transferable element located in pOLA52. To test this possibility, we performed PCR screening to determine if pOLA52 specific DNA sequences were prevalent among the test plasmids. To our surprise, however, none of the plasmids that carried *oqxAB* and *aac(6*′*)Ib-cr* contained such sequences of pOLA52 (Data not shown).

### CONTRIBUTION OF *oqxAB* AND *aac(6*′*)Ib-cr* TO THE ELEVATED CIPROFLOXACIN MIC IN *S. typhimurium*

To directly prove the degree of contribution of *oqxAB* and *aac(6*′*)Ib-cr* to the development of fluoroquinolone resistance in *S. typhimurium*, *oqxAB* was cloned into a pTrc expression vector and transformed into *S. typhimurium* LT2 strain. Compared to the original *oqxAB* negative *S. typhimurium* LT2 strain, pTrc-*oqxAB-* carrying *S. typhimurium* LT2 exhibited a CIP MIC of 0.25 μg/ml, with a 20-fold increase. However, *S. typhimurium* LT2 carrying pTrc-*oqxAB* showed much weaker growth than its parental counterpart, which was presumably due to the fitness cost caused by the over-expression of *oqxAB* in the host strain. To overcome this problem, the plasmids that carried *oqxAB* and *aac(6*′*)Ib-cr* were extracted from different clinical *S. typhimurium* isolates and electroporated into *S. typhimurium* LT2 with no success. The plasmids were then electroporated into an *oqxAB*-negative *S. typhimurium* strain 11–28. Upon acquisition of such plasmid, the CIP MIC of this *S. typhimurium* strain increased by approximately fourfold (**Table 2**). To further prove the contribution of *oqxAB* and *aac(6*′*)Ib-cr* to *S. typhimurium* fluoroquinolone resistance, the plasmid carrying such genes in a clinical *S. typhimurium* strain 10–63 was cured and it showed that the curing of the plasmid in 10–63 decreased the CIP MIC by approximately fourfold (**Table 2**). Taken together, our data had proven that *oqxAB* and *aac(6*′*)Ib-cr* contributed to about four fold increase of CIP MIC in *S. typhimurium*. The MICs of other antibiotics were also determined for *S. typhimurium* that acquired *oqxAB*, *aac(6*′*)Ib-cr* encoding plasmids. In addition, it is showed that acquisition of *oqxAB* and *aac(6*′*)Ib-cr* encoding plasmids ensured resistance to ampicillin, chloramphenicol, streptomycin, nalidixic acid, sulfamethoxazole, tetracycline, trimethoprim, and olaquindox in addition to the elevated CIP MIC (**Table 2**). This is also consistent to our previous finding that the presence of *oqxAB* in *S. typhimurium* was associated with the ACSSuT R phenotype. As much as 56% of *oqxAB*-positive *S. typhimurium* clinical isolates were resistant to ACSSuT, whereas only 14% of *oqxAB*-negative isolates were resistant to ACSSuT (Wong et al., 2013).

**Table 2:** MIC profiles for *Salmonella* strains with various *oqxAB* and *aac(6*′*)Ib-cr* -borne plasmids.

Strain	MIC (μg/ml)										
	AMP	CRO	CIP	NA	TET	CHL	SUL	TRI	AMK	GEN	OLA	STE
11–28*	≤4	≤1	0.012	4	2	≤4	≤128	≤4	≤4	≤1	8	8
P06–57#	≥128	≤1	0.05	16	64	≥128	≥1024	32	≤4	32	128	≥128
P07–43#	≥128	≤1	0.05	32	64	≥128	≥1024	32	≤4	32	64	≥128
P08–11#	≥128	≤1	0.05	16	64	≥128	≥1024	32	≤4	32	64	≥128
P10–9#	≥128	≤1	0.05	16	64	≥128	≥1024	32	≤4	32	128	≥128
10–63*	≥128	≤1	1	≥128	64	≥128	≥1024	32	≤4	≤1	512	32
10–63C	16	≤1	0.25	≥128	2	≥128	≥1024	≤4	≤4	≤1	16	8

AMP, ampicillin; CRO, ceftriaxone; CIP, ciprofloxacin; NA, Nalidixic acid; TET, tetracycline; CHL, chloramphenicol; SUL, sulfamethoxazole; TRI, trimethoprim; AMK, amikacin; GEN, gentamicin; STE, streptomycin; OLA, olaquindox.

^*^Salmonella clinical isolates with various *oqxAB*, *aac(6*′*)Ib-cr* background; #transformants with the transformation of *oqxAB*, *aac(6*′*)Ib-cr* encoding plasmid from different clinical *Salmonella* isolates to parental *Salmonella* strain 11–28; C, *oqxAB*, *aac(6*′*)Ib-cr* encoding plasmid cured strain.

### CONTRIBUTION OF *oqxAB* AND *aac(6*′*)Ib-cr* TO ELEVATED MPC OF FLUOROQUINOLONE IN *S. typhimurium*

To validate the hypothesis that *oqxAB* and *aac(6*′*)Ib-cr* contributed to mutation development, MPC of CIP were determined for *S. typhimurium* with and without *oqxAB*. As shown in **Table 3**, *oqxAB, aac(6*′*)Ib*-*cr* positive clinical *Salmonella* isolates, 06–57, 07–43, and 08–11 exhibited much higher MPC of CIP than the *oqxAB, aac(6*′*)Ib*-*cr*-negative *Salmonella* strains, 05–41, 07–54, and 10–25 (**Table 3**). Furthermore, although *Salmonella* 11–28 strain exhibited MPC for CIP of about 0.1 μg/ml, transformation of plasmids from other clinical *Salmonella* isolates carrying *oqxAB*, *aac(6*′*)Ib*-*cr* to *Salmonella* 11–28 dramatically increased its MPC to 2∼4 μg/ml. On the other hand, *Salmonella* 10–63 exhibited MPC of 8 μg/ml, yet the curing of the *oqxAB*, *aac(6*′*)Ib*-*cr* encoding plasmid led to a slightly decreased MPC (4 μg/ml). The minimal effect of curing of *oqxAB*, *aac(6*′*)Ib*-*cr* encoding plasmid on the MPC of 10–63 may be due to the fact that the long-term starvation stress used to cure the plasmid may have caused stress response to develop in the isolate, thereby indirectly contributing to the elevated MPC for strain.

**Table 3:** MICs of nalidixic acid (NA) and ciprofloxacin (CIP) and mutation prevention concentration (MPC) toward CIP of *Salmonella* isolates with various background of *oqxAB* and *aac(6*′*)Ib-cr*.

*Salmonella* Isolate	*oqxAB*,*aac(6*′*)Ib-cr*	QRDR Mutations		MIC (μg/ml)		MPC (μg/ml)	GyrA mutation^†^	MPC/MIC
		GyrA	ParC	NA	CIP	CIP		
06–57*	+	WT	WT	32	0.1	2	NT	20
07–43*	+	WT	WT	16	0.05	0.5	NT	10
08–11*	+	WT	WT	32	0.1	1	NT	10
05–41*	–	D87N	WT	32	0.025	0.1	NT	4
07–54*	–	WT	WT	16	0.012	0.1	NT	8
10–25*	–	WT	WT	4	0.025	0.1	NT	4
11–28*	–	WT	WT	4	0.012	0.1	WT	8
p06–57#	+	WT	WT	16	0.05	4	D87N	80
p07–43#	+	WT	WT	32	0.05	2	WT	40
p08–11#	+	WT	WT	16	0.05	2	D87G	40
p10–9#	+	WT	WT	16	0.05	2	WT	40
10–63*	+	D87N	WT	≥128	1	8	D87Y	8
10–63c	–	D87N	WT	≥128	0.25	4	D87Y	16

NA, Nalidixic acid; CIP, ciprofloxacin.

*Salmonella clinical isolates with various *oqxAB*, *aac(6*′*)Ib-cr* background; #transformants with the transformation of *oqxAB*, *aac(6*′*)Ib-cr* encoding plasmids from different clinical *Salmonella* isolates to parental *Salmonella* strain 11–28; ^†^GyrA mutation from strains that were selected after MPC assay and have CIP MIC between 0.5 and 4 μg/ml; c, *oqxAB*, *aac(6*′*)Ib-cr* encoding plasmid cured strain; NT, Not tested.

Ten to 63 C. It has been shown that long-term starvation stress stimulates the stringent SOS response in bacteria, which is essential in bacteria for acquisition of mutations leading to resistance to some antibiotic drugs (Fung et al., 2010). Most importantly, compared to *Salmonella* 11–28 alone, which did not develop *gyrA* mutation in MPC assay, *Salmonella* 11–28 transformed with *oqxAB*, *aac(6*′*)Ib*-cr encoding plasmids from *Salmonella* 06–57 and 08–11 developed single mutation in *gyrA*, which may partly contributed to the increase of CIP MPC (**Table 3**). It is probably due to that the presence of *oqxAB* and *aac(6*′*)Ib*-cr may enable *S. typhimurium* to survive under fluoroquinolone stress and facilitate subsequent development of target mutations. Nevertheless, these data confirm that *oqxAB, aac(6*′*)Ib*-*cr* plays a key role in elevated CIP MIC and MPC, and hence resistance to fluoroquinolone in *S. typhimurium*.

## DISCUSSION

An important finding in this work is that the *oqxAB* and *aac(6*′*)Ib-cr* gene products not only directly contribute to the elevated CIP MIC, but also enhance the ability of *S. typhimurium* to survive in an environment with high dose of CIP, which may in turn facilitate the development of fluoroquinolone resistance. The mechanism of fluoroquinolone resistance in *Salmonella* has conventionally been attributed to double mutations in *gyrA* with or without a single *parC* mutation (Casin et al., 2003; Chu et al., 2005). Unlike *E. coli* and *Campylobacter*, double *gyrA* mutations in *Salmonella* were rare and presumably difficult to acquire, therefore fluoroquinolone remained an effective treatment of choice for severe *Salmonella* infections. In this study, we demonstrated that acquisition of the *oqxAB or oqxAB, aac(6*′*)Ib-cr* genes in *S. typhimurium*, could mediate development of resistance to CIP (CIP MIC ≥1 μg/ml). We postulate that the pump activities and enzymatic hydrolysis of fluoroquinolones enable the organisms to withstand antibiotic pressure for a prolonged period, during which mutational changes can occur. Elevation of the antibiotic resistance potential of *Salmonella* is one way by which *oqxAB* can help the host strain to successfully launch clinical infection in human, leading to a dramatic increase in the proportion of *oqxAB* positive strains observable among clinical *Salmonella* isolates recovered in recent years (Wong et al., 2014). The increased prevalence of *oqxAB* positive *S. typhimurium* in clinical isolates also contributes directly to a higher percentage of fluoroquinolone resistance in clinical salmonella strains. In 2011, the proportion of the *oqxAB* positive *S. typhimurium* in Hong Kong that were found to be resistant to CIP reached 34% (Data not shown).

The fact that *oqxAB* could not be found in *S. typhimurium* until 2006 may be due to its poor ability to replicate in *Salmonella* initially; this notion is supported by the fact that transformation of *oqxAB*-borne plasmid to *S. typhimurium* did not elevate MIC of CIP in these strains and that direct expression of *oqxAB* into *S. typhimurium* had a fitness cost in this work (Hansen et al., 2007; Wong et al., 2013). Nevertheless, our data indicate that the *oqxAB* gene has adapted to co-exist in *S. typhimurium*. In this study, *oqxAB* were found to be associated with IS*26* but not carried by pOLA52-like plasmids, suggesting *oqxAB* was excised from pOLA52 and integrated into other plasmids mediated by IS*26* transposase. Since no *oqxAB* encoding plasmid in *Salmonella* has been sequenced, the mechanism underlying the co-existence of *oqxAB* and *aac(6*′*)Ib-cr* in over 80% of the *oqxAB*-positive strain is not clear. The quick expansion of *oqxAB* and *aac(6*′*)Ib-cr* positive, CIP-resistant *S. typhimurium* will pose huge threat to efforts of infection control of *Salmonella* infections. Urgent actions are required to halt further transmission of the *oqxAB* positive strains in both environmental and clinical settings. In addition, it remains to be seen if *oqxAB* has been taken up by other bacterial species and whether it plays a role in the evolution of resistance and virulence traits of various bacterial pathogens. Findings in this work also highlight a need to investigate the impact of *oqxAB* in a wide range of foodborne and zoonotic pathogens.

## Conflict of Interest Statement

The authors declare that the research was conducted in the absence of any commercial or financial relationships that could be construed as a potential conflict of interest.
